# Retraction: Effect of platelet‐rich plasma in treating patients with burn wounds: A meta‐analysis

**DOI:** 10.1111/iwj.70018

**Published:** 2024-08-09

## Abstract

Lin C, Xin L, Xie S. Effect of platelet‐rich plasma in treating patients with burn wounds: a meta‐analysis. *Int Wound J*. 2023; 21(3): e14486. doi: 10.1111/iwj.14486.

The above article, published online on 06 November 2023, in Wiley Online Library (http://onlinelibrary.wiley.com/), has been retracted by agreement between the journal Editor in Chief, Professor Keith Harding; and John Wiley & Sons, Ltd. It came to the publisher's attention from a third party that a number of articles shared concerning similarities in format and structure. Following an investigation by the publisher, the retraction of this article has been agreed on because the peer review and publishing process for this article were found to have been manipulated. The authors did not respond to our notice of retraction.

Lin C, Xin L, Xie S. Effect of platelet‐rich plasma in treating patients with burn wounds: a meta‐analysis. *Int Wound J*. 2023; 21(3): e14486. doi: 10.1111/iwj.14486.

The above article, published online on 06 November 2023, in Wiley Online Library (http://onlinelibrary.wiley.com/), has been retracted by agreement between the journal Editor in Chief, Professor Keith Harding; and John Wiley & Sons, Ltd. It came to the publisher's attention from a third party that a number of articles shared concerning similarities in format and structure. Following an investigation by the publisher, the retraction of this article has been agreed on because the peer review and publishing process for this article were found to have been manipulated. The authors did not respond to our notice of retraction.

